# Detection of Adulteration and Pesticide Residues in Chinese Patent Medicine Qipi Pill Using KASP Technology and GC-MS/MS

**DOI:** 10.3389/fnut.2022.837268

**Published:** 2022-03-10

**Authors:** Gang Wang, Xuanjiao Bai, Xiaochen Chen, Ying Ren, Xiaohui Pang, Jianping Han

**Affiliations:** Institute of Medicinal Plant Development, Chinese Academy of Medical Sciences & Peking Union Medical College, Beijing, China

**Keywords:** Chinese patent medicines, adulteration, pesticide residues, KASP technology, DNA mini-barcode, GC-MS/MS

## Abstract

Chinese patent medicines (CPMs) are of great value for the prevention and treatment of diseases. However, adulterants and pesticide residues in CPMs have become the “bottleneck” impeding the globalization of traditional Chinese medicine. In this study, 12 batches of commercially available Qipi pill (a famous CPM recorded in Chinese Pharmacopeia) from different manufacturers were investigated to evaluate their authenticity and quality safety. Considering the severely degraded DNA in CPMs, kompetitive allele specific PCR (KASP) technology combined with DNA mini-barcodes was proposed for the quality regulation of a large number of products in CPM market. The residues of four kinds of pesticides including pentachloronitrobenzene (PCNB), hexachlorocyclohexane (HCH), aldrin, and dichlorodiphenyltrichloroethane (DDT) were quantified using gas chromatography and tandem mass spectrometry (GC-MS/MS). The results indicated that in two of the 12 batches of Qipi pill, the main herbal ingredient *Panax ginseng* was completely substituted by *P. quinquefolius*, and one sample was partially adulterated with *P. quinquefolius*. The PCNB residue was detected in 11 batches of Qipi pill, ranging from 0.11 to 0.46 mg/kg, and the prohibited pesticide HCH was present in four samples. Both adulteration and banned pesticides were found in two CPMs. This study suggests that KASP technology combined with DNA mini-barcodes can be used for the quality supervision of large sample size CPMs with higher efficiency but lower cost. Our findings also provide the insight that pesticide residues in CPMs should be paid more attention in the future.

## Introduction

Chinese patent medicines (CPMs) play a vital role in health care and disease prevention and treatment in China (1, 2). However, the frequent adulteration of original materials has become a great challenge for the modernization of CPMs (3). A previous survey on the authenticity of herbal medicines from China's markets found that ~4.2% of 1,260 tested samples were adulterated (4). It will be extremely challenging to identify their botanical origin by traditional methods based on morphological and microscopic characteristics especially when the medicinal ingredients are pulverized into superfine powder and made into CPMs. A previous study found that in 33 root samples and 70 powder samples of *Withania somnifera* (L.) Dunal, 23% were identified to be counterfeit, among which 22% of the non-authentic samples were powder, suggesting that the adulteration was more prevalent in processed products (5). The incorporation of adulterants will undoubtedly lead to a decline in the efficacy of CPMs (6–8). Additionally, the excessive pesticides within herbs in CPMs reflect another enormous threat on clinical use (9, 10). The determination of 17 organochlorine pesticides in 86 batches of *Panax ginseng* C. A. Mey showed that 55.8% of the samples contained pentachloronitrobenzene beyond the upper limit (11). The adulteration and substitution of original materials, which weakens their pharmacological function, and these chemical pesticides processed together with medicinal herbs into various CPMs, may pose a potential risk to the health and safety of end users (9). Therefore, it is imperative to investigate the authenticity and quality safety of CPMs in market.

Chemical profiling techniques have always played an indispensable role in the quality control and standardization of medicinal materials and CPMs (12–14). However, detection of adulteration and substitution of some herbs containing similar active chemical components, such as *P. ginseng* and *Panax quinquefolius* L., may not be accomplished only by analyzing the presence or absence of specific chemical constituents (15–17). DNA barcoding has been proposed as a powerful tool for the authentication of raw herbal materials (18). However, DNA degrades seriously when plant materials are crushed and processed into tablets, pills, capsules and other preparations. At this point, conventional DNA barcodes may not work well (19). Meusnier et al. suggested the use of “mini-barcode” to solve this problem well. It was indicated that the full-length cytochrome c oxidase subunit 1 (*COI*) barcode of 650 bp was able to identify 97% of the tested species, while the identification success rates by using the shorter sequences of 100 and 150 bp within *COI* region were also up to 90 and 95%, respectively (20, 21). Further, nucleotide signatures with generally 20–50 bp were utilized to test specific components in DNA-degraded materials (22–24). And the KASP (kompetitive allele specific PCR) technology represents a new type of DNA polymorphism assay, which can identify the InDels (insertion/deletion of one or more nucleotides) and SNPs (single nucleotide polymorphisms) at specific genomic loci. With the advantages of low cost, high throughput, and high specificity and sensitivity, KASP technology has been widely accepted in gene localization, molecular marker-assisted breeding, germplasm resources identification, etc (25–27). The feature of high-throughput of KASP could help realize the goal of detecting thousands of samples each time. Furthermore, there is no need to synthesize specific fluorescent probes for different SNP sites, which also makes this method both efficient and cost-effective. Therefore, we hypothesized that it may be also of great potential in the mass testing of CPM quality.

Qipi pill, recorded in Chinese Pharmacopeia 2020, is a widely used patent medicine to strengthen spleen and harmonize stomach. It is made from 11 medicinal materials (Ginseng Radix et Rhizoma, Atractylodis Macrocephalae Rhizoma, Poria, Glycyrrhizae Radix et Rhizoma, Citri Reticulatae Pericarpium, Dioscoreae Rhizoma, Nelumbinis Semen, Crataegi Fructus, medicated leaven, Hordei Fructus Germinatus, and Alismatis Rhizoma) supplemented with honey. Among them, *P. ginseng* (Ginseng Radix et Rhizoma) is regarded as the monarch drug, which is often adulterated or substituted by *P. quinquefolius*. In this study, a total of 12 batches of Qipi pill from different manufacturers were investigated to provide a theoretical reference for the market surveillance of CPMs. The KASP technology combining with DNA mini-barcoding was put forward for the first time to identify *P. ginseng* in Qipi pill. Besides, the pesticide residues of pentachloronitrobenzene (PCNB), hexachlorocyclohexane (HCH), aldrin and dichlorodiphenyltrichloroethane (DDT) in CPMs were quantified by gas chromatography-tandem mass spectrometry (GC-MS/MS). Detailed information is available below.

## Materials and Methods

### Collection of the CPM Products Qipi Pill

A total of 12 batches of Qipi pill were collected from the markets in Beijing, Tianjin, Shenyang, Xi'an, Chengde, Lanzhou, and Anguo of China. All the products were honeyed pills. The detailed information of these samples is listed in Table 1.

**Table 1:** Sample information of Qipi pill in this study.

**Sample no**.	**Type**	**Source**	**Ingredients on label**	**Identification result**
QPW1	Honeyed pill	Beijing	*P. ginseng*	*P. ginseng*
QPW2	Honeyed pill	Tianjin	*P. ginseng*	*P. ginseng*
QPW3	Honeyed pill	Xi'an	*P. ginseng*	*P. quinquefolius*
QPW4	Honeyed pill	Shenyang	*P. ginseng*	*P. ginseng*, *P. quinquefolius*
QPW5	Honeyed pill	Lanzhou	*P. ginseng*	*P. ginseng*
QPW6	Honeyed pill	Chengde	*P. ginseng*	*P. quinquefolius*
QPW7	Honeyed pill	Beijing	*P. ginseng*	*P. ginseng*
QPW8	Honeyed pill	Beijing	*P. ginseng*	*P. ginseng*
QPW9	Honeyed pill	Beijing	*P. ginseng*	*P. ginseng*
QPW10	Honeyed pill	Beijing	*P. ginseng*	*P. ginseng*
QPW11	honeyed pill	Beijing	*P. ginseng*	*P. ginseng*
QPW12	Honeyed pill	Anguo	*P. ginseng*	*P. ginseng*

### Chemicals and Standards

QuEChERS extraction salt packets consisting of 6 g of anhydrous magnesium sulfate (MgSO_4_) and 1.5 g of sodium acetate, and QuEChERS cleanup tubes containing 1.2 g of anhydrous MgSO_4_, 0.4 g of primary secondary amine (PSA) and 0.4 g of octadecylsilyl (C18) were purchased from Qingyun Experimental Consumables Co., Ltd. (Shandong, China). The HPLC-grade acetonitrile (MeCN) and acetic acid were obtained from CNW Technologies GmbH (Duesseldorf, Germany). The pesticide mixture standards of PCNB, hexachlorocyclohexane (α-HCH, β-HCH, and δ-HCH), aldrin and dichlorodiphenyltrichloroethane (p,p′-DDE, p,p′-DDD, o,p′-DDT, and p,p′-DDT) were purchased from Alta Scientific Co., Ltd. (Tianjin, China). The reference pesticides were diluted using ethyl acetate to achieve the concentrations of 20, 50, 100, 200, and 500 ng/mL, respectively. The stock multi-standard solutions were stored at −20°C until they were used for further analyses.

### DNA Extraction, PCR Amplification, and Sequencing

Approximately 50 mg of the pills were added to a centrifuge tube and six parallel tubes of each sample were prepared for the DNA extraction of CPMs. The samples were ground into fine powder in a ball-milling machine (Retsch Co., Germany) at a frequency of 30 Hz for 2 min. Seven hundred microliter of pre-wash buffer (700 mM NaCl; 100 mM Tris-HCl, pH 8.0; 20 mM EDTA, pH 8.0; 2% PVP-40 and 0.4% β-mercaptoethanol) was added to the tubes to wash the sample powder several times until the supernatant was colorless and transparent, and then the mixture was centrifuged at 7,500 rpm for 5 min. The genomic DNA was extracted from the precipitate using the Plant Universal Genomic DNA Extraction Kit (Tiangen Biotech Beijing Co.) in accordance with the manufacturer's instructions. Finally, the DNA in six duplicate tubes was eluted with double-distilled water into a single tube.

The specific primers 4F (TGCAGAATCCCGTGAACC)/4R (GCCAAGGACTCGCATTTG) were used to amplify the short fragments within ITS2 region of *Panax* referring to the study of Liu et al. (22). The polymerase chain reaction (PCR) was performed in a 25-μl system consisting of 12.5 μl of 2 × PCR Master Mix (Aidlab Biotechnologies Co., Ltd.), 1.0 μL of forward/reverse primers (2.5 μM), 1.0 μL of DNA templates, and double-distilled water. The reactions were performed in a thermal cycler (Applied Biosystems Co., USA) with the following program: 94°C for 5 min; 35 cycles of denaturation at 94°C for 30 s, annealing at 53°C for 30 s and elongation at 72°C for 45 s; and a final extension at 72°C for 10 min. The PCR products were examined *via* 1% agarose gel electrophoresis and purified for the bidirectional sequencing with the primer pair 4F/4R based on the Sanger sequencing method using an ABI 3730XL sequencer (Applied Biosystems Co., USA).

The DNA sequencing results were edited and assembled via CodonCode Aligner 5.2.0 (CodonCode Co., USA). The low-quality regions were removed from both ends of the raw sequences, and consensus sequences were finally generated. BLAST analysis of the DNA mini-barcode sequences was performed in the website of the National Center for Biotechnology Information (NCBI, https://www.ncbi.nlm.nih.gov/). Then the sequencing peak figures were aligned using CodonCode Aligner software to find the possible adulteration in CPMs.

### KASP Assay

According to the previous study (28), two mutation loci within the DNA mini-barcode region were selected as the KASP markers to distinguish *P. ginseng* from *P. quinquefolius*. The KASP primers were designed based on the stable SNPs via Kraken™ software (Table 2). The KASP assay was performed in a PCR system consisting of 0.778 μL of KASP master mix (LGC Biosearch Technologies, UK), 0.022 μL of KASP assay mix, and 0.8 μL of DNA templates at the concentration of 5–10 ng/μL. KASP reactions were carried out in a Nexar/Sollex/Araya platform (LGC Biosearch Technologies, UK) with the following thermal cycling conditions: 94°C for 15 min; 94°C for 20 s, 61–55°C for 1 min (dropping 0.6°C per cycle for 10 cycles); followed by 26 cycles of 94°C for 20 s and 55°C for 1 min. Finally, the fluorescence signal was analyzed using SNPViewer software.

**Table 2:** Primer sequences for KASP assay in this study.

**Primer name**	**Primer_AlleleX (5′-3′)**	**Primer_AlleleY (5′-3′)**	**Primer_Common (5′-3′)**
PAN_SNP1	CCCCCAACCCATCACTCCT	CCCCCCAACCCATCACTCCC	CTCCGCCTCRACTCCCGCAA
PAN_SNP2	CCGCCCCTCCGCCTCG	ATCCGCCCCTCCGCCTCA	CCCCCCAACCCATCACTCCYTT

### Determination of Pesticide Content in Qipi Pills Based on GC-MS/MS

#### Sample Preparation

A 2.0 g aliquot of each CPM sample was weighed and placed into a 50 mL centrifuge tube, and 10 mL of water were added. The samples were thoroughly shaken and mixed, and stood for 30 min. Then 15 mL of acetonitrile with 1% of acetic acid, 6 g of anhydrous magnesium sulfate and 1.5 g of sodium acetate were added into the tubes, and the centrifugation was carried out at 4,200 rpm for 5 min after 1 min of violent oscillation. The supernatant was transferred to a new centrifuge tube containing anhydrous MgSO_4_ (1.2 g), PSA (0.4 g), and C18 (0.4 g). The mixture was shaken for 1 min and then centrifuged at 4,200 rpm for 5 min. Five milliliter of the supernatant were accurately added to another tube and blown dry with nitrogen in a 40 °C water bath. Finally, the sample was redissolved by 1 mL of ethyl acetate and then filtered through microporous membranes for the subsequent quantitation.

#### Analytical Conditions

The content analysis of pesticide residues was performed using a GC-MS/MS system Agilent 7000D (Agilent Technologies, USA). A HP-5MA Ultra Inert capillary chromatographic column (15 m × 0.25 mm × 0.25 μm) was utilized for the GC separations. The temperature of the column was as follows: 40°C for 1 min, 40°C/min to 120°C, 5°C/min to 240°C, and 12°C/min to 300°C for 6 min. Nitrogen (purity ≥ 99.999%) was used as the carrier gas with the flow rate of 1.0 mL/min. The injection port temperature and transfer-line temperature were 280 °C. The injection volume in unshunted mode was 1 μL. The analysis in mass spectrometer was under MRM mode and the electron impact ion source was at 70 eV. The ion source temperature was maintained at 280 °C. The solvent delay was for 4 min.

## Results

### DNA Extraction and Amplification of DNA Mini-Barcodes From CPMs

**Figure 1:**
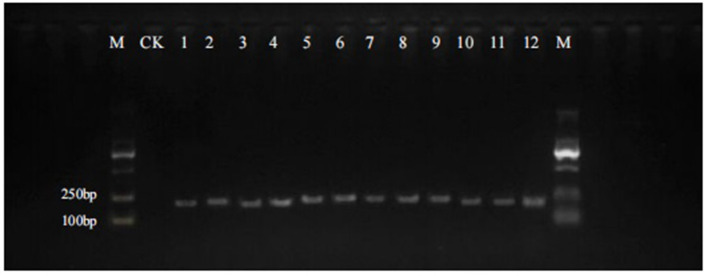
PCR amplification of 12 batches of Qipi pill with the primer pair 4F/4R. M, DNA marker; CK, negative control; 1–12, samples of QPW1–QPW12.

The quality of the extracted DNA from 12 batches of Qipi pills was measured by the spectrophotometer NanoDrop 2000, and the value of A_260/280_ ranged from 1.8 to 2.0, indicating that the DNA quality met the requirements of subsequent experiments. The DNA mini-barcode within ITS2 region of *Panax* was amplified with the primers 4F/4R, and the agarose gel electrophoresis of PCR products was used to examine the success rate of the amplification for target short fragments (Figure 1). The electrophoresis strips showed that all the 12 samples were successfully amplified, which also proved the excellent applicability of the primers. The size of the obtained target segments was about 160 bp, which was in line with the expectation of DNA mini-barcodes. Then the PCR products were purified and bidirectionally sequenced based on Sanger sequencing method.

### Identification of *P. ginseng* in Qipi Pill Based on DNA Mini-Barcoding

**Figure 2:**
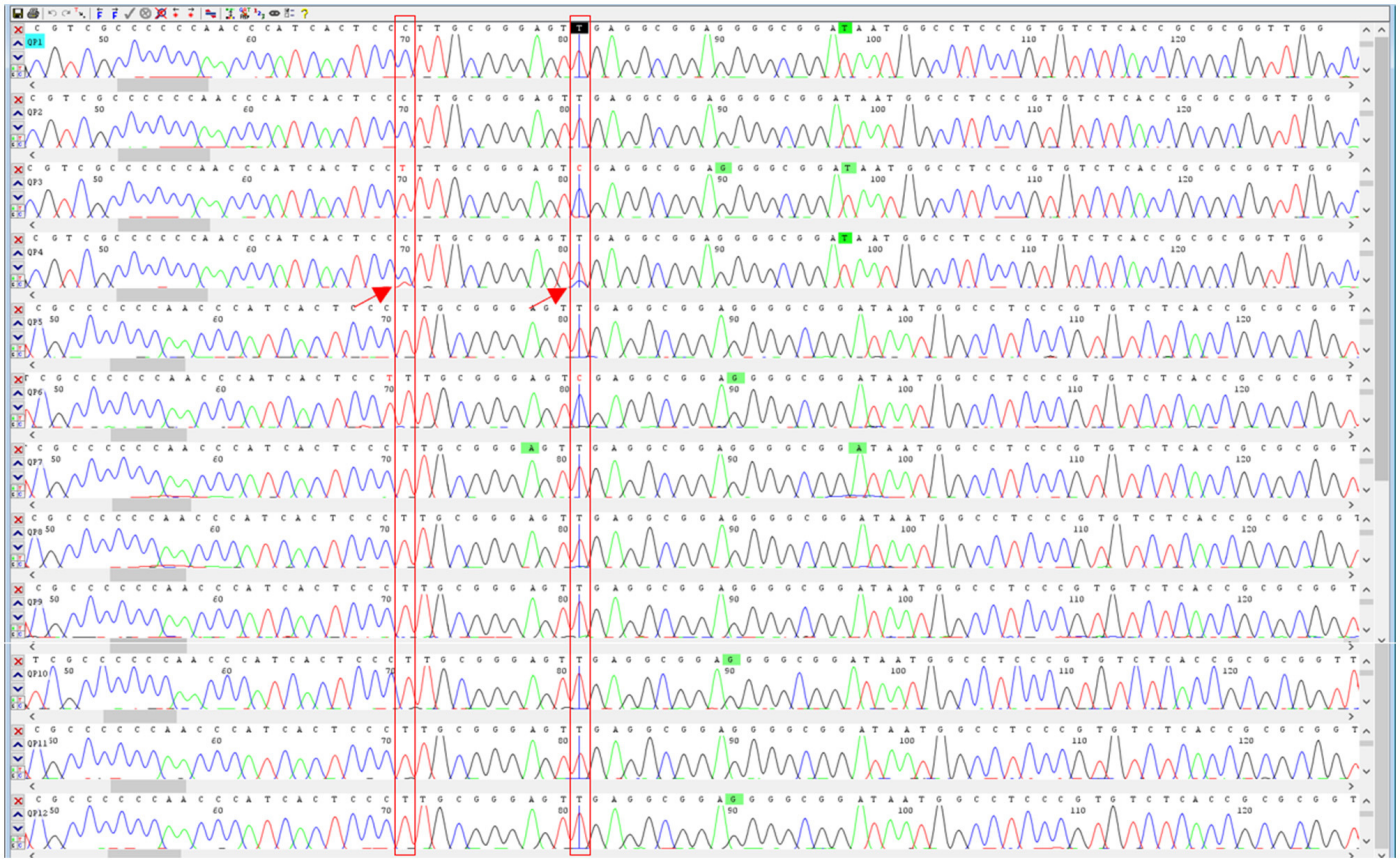
Sequencing peak profiles of the 12 samples of Qipi pill. The double peaks marked by red arrows indicated adulterated *P. quinquefolius* in the corresponding CPM products.

The DNA mini-barcodes within ITS2 region were applied to determine whether the CPM products contained the labeled component *P. ginseng*, and the sequencing peak figures were utilized to discriminate the possible adulteration according to the previous studies of (22, 28). After shearing and assembling of the DNA sequencing data, 12 sequences of 159 bp were finally obtained for Qipi pills. The BLAST results of the sequences from NCBI indicated that *P. ginseng* was detected in 10 samples (QPW1, QPW2, QPW4, QPW5, QPW7, QPW8, QPW9, QPW10, QPW11, and QPW12), while *P. quinquefolius*, which was not listed on the label, was found in the remaining two samples QPW3 and QPW6 (Table 1). Based on the two stable SNP loci within ITS2 regions, double peak method was carried out to analyze the mixture ratio of *P.ginseng* and *P.quinquefolius*. By analyzing SNPs in the sequencing peak diagrams, it was inferred that *P. ginseng* in QPW3 and QPW6 was completely substituted by *P. quinquefolius*. The sample QPW4 showed double peaks at the SNP sites of 70 and 81 bp (Figure 2). It could be concluded that *P. quinquefolius* was adulterated in the raw materials of this product, and *P. ginseng* still possessed a dominant ratio according to the height of the peaks. Taken together, in the 12 CPM products, the principal herb *P. ginseng* in two batches was thoroughly substituted with *P. quinquefolius*, and partially adulterated in one batch.

### Application of KASP Technology in the Quality Control of CPMs

**Figure 3:**
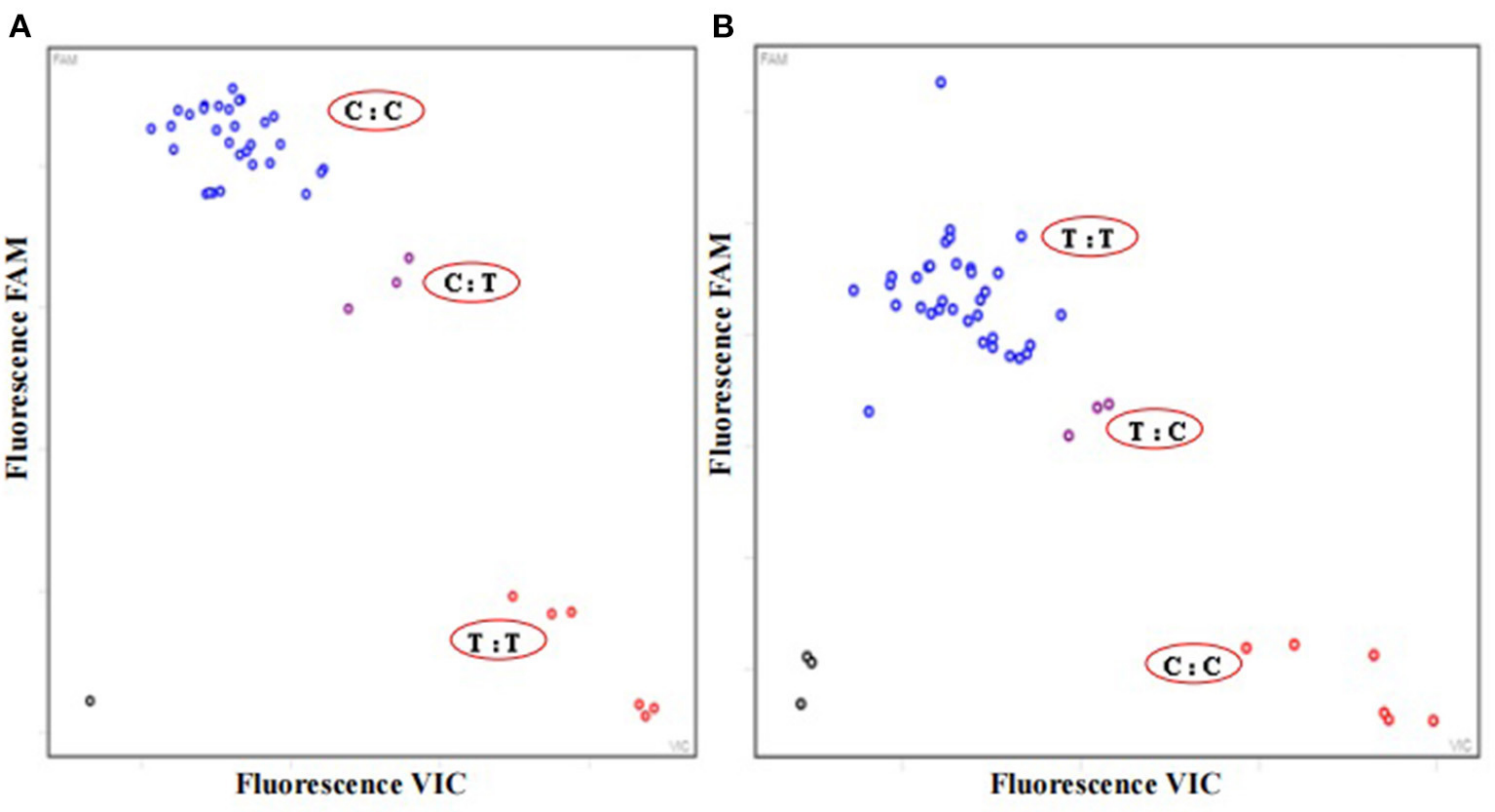
Results of KASP assay for SNP 1 **(A)** with the primer group PAN_SNP1 and SNP 2 **(B)** with PAN_SNP1 for 12 batches of Qipi pill. Three replicates were set for each sample. The horizontal and vertical axes show the fluorescence signal of bases at different SNP sites. The blue and red circles represent the fluorescence signal of *P. ginseng* and *P. quinquefolius* respectively, and the purple signal represent the simultaneous appearance of *P. ginseng* and *P. quinquefolius*. The black circles on the bottom left are no-template control. 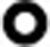 Negative control; 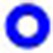 *P. ginseng*: QPW1, QPW2, QPW5, and QPW7-QPW12; 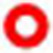 *P. quinquefolius*: QPW3 and QPW6; 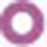 *P. ginseng* and *P. quinquefolius*: QPW4.

Based on the two stable SNP sites in the ITS2 mini-barcode region of *P. ginseng* and *P. quinquefolius*, two sets of primers were designed for KASP assay to investigate the adulteration and substitution in the 12 batches of Qipi pills. The fluorescence signal information reflected the herbal composition in different CPM products. The identification results of the first and second SNPs were showed in Figures 3A,B, respectively. It was indicated that the fluorescence signal of *P. ginseng* was captured in the nine CPM samples, i.e., QPW1, QPW2, QPW5, QPW7, QPW8, QPW9, QPW10, QPW11, and QPW12, of which the fluorescence signals also gathered together. Meanwhile, *P. quinquefolius* alone was detected in QPW3 and QPW6. Moreover, the signal of QPW4 showed the coexistence of *P. ginseng* and *P. quinquefolius* in this CPM product (Figure 3). In conclusion, in three batches of Qipi pill, the raw material of *P. ginseng* was adulterated or completely substituted by *P. quinquefolius*, which was consistent with the identification results of Sanger sequencing.

### Investigation of Pesticide Residues in CPMs

**Figure 4:**
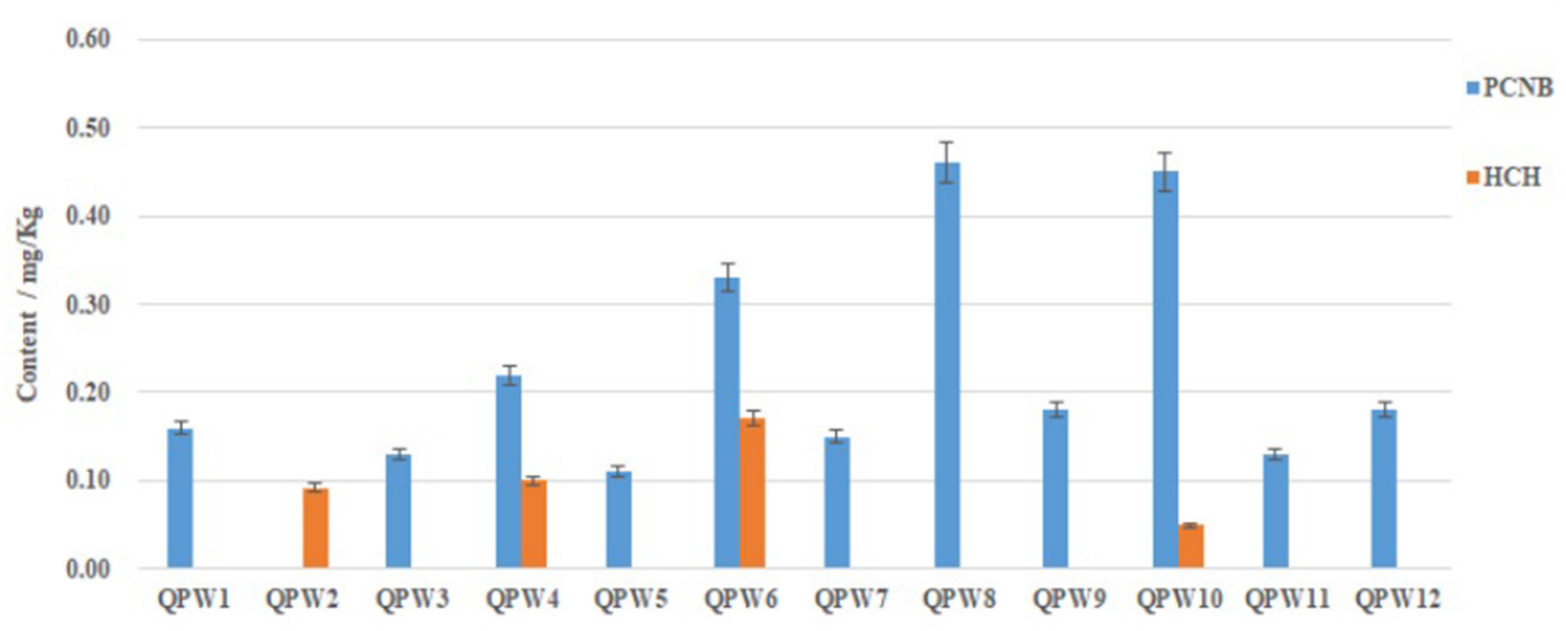
Contents of pesticide residues in Qipi pills. DDT and Aldrin were not detected in all the 12 CPM samples.

The linear range of the GC/MS-MS determination was from 20 to 500 ng/mL, and the correlation coefficients (*R*^2^) were all higher than 0.9992, with the recovery rate of 84.3–104.2%. The minimum detection limit was 0.01 mg/kg (Supplementary Figure 1). Under the set analytical conditions, the trueness and precision met the detection requirements. The residues of four kinds of pesticides in the 12 batches of Qipi pill were showed in Figure 4. Neither the pesticide DDT nor aldrin was detected in all the samples. The banned pesticide HCH was absent in eight samples, while its content in the remaining four samples (QPW2, QPW4, QPW6, and QPW10) ranged from 0.049 to 0.17 mg/kg. In addition, PCNB was commonly identified in 11 batches of Qipi pill, with the content ranging from 0.11 mg/kg to 0.46 mg/kg. Among them, the PCNB content in QPW6, QPW8, and QPW10 were 0.33, 0.46, and 0.45 mg/kg, respectively, which were significantly higher than those in the other samples. HCH and PCNB were simultaneously detected in samples QPW4, QPW6, and QPW10. Together these results from composition analysis with KASP and pesticide leftover detection by GC/MS-MS indicated that both banned pesticides and herbal adulteration existed in the two CPM products QPW4 and QPW6.

## Discussion

### Application of DNA Mini-Barcoding in the Identification of Herbal Materials in CPMs

Reliable plant source of raw materials is the foundation of the clinical efficacy of CPMs. However, identifying the components of medicinal herbs by conventional methods is always challenging when they are crushed and mixed with auxiliary materials into various dosage forms. DNA barcoding technology has been authorized as a standard method for the identification of traditional Chinese medicines in Chinese Pharmacopeia (29). It has been widely applied to the authentication of medicinal materials, decoction pieces, seeds and seedlings, and proprietary Chinese medicine (18, 30, 31). This method is basically not susceptible to external factors. However, most botanic materials go through a series of processing procedures before they are produced into diverse preparations of proprietary Chinese medicines. Thus badly degraded DNA may result in an inability to be extracted and amplified as complete DNA barcode sequences (32). Under such circumstances, using specific primers to obtain shorter DNA segments for the identification of herbal ingredients in CPMs presents greater advantage (33). A previous study suggested that the DNA fragments ranging from 88 to 121 bp could be successfully amplified from pulverized samples boiled for 60 min, but the longer sequences failed (34). The development of DNA mini-barcoding enables its wider applications in the composition analysis of CPMs. Combining with the double peak method based on SNPs, it can also help detect the incorporation of non-label ingredients (28). Xin et al. (35) successfully identified the multiple ingredients in the TCM formula Jiuwei Qianghuo pill using DNA barcoding and single molecule real-time sequencing. Dong et al. (36) searched the variable regions from the whole chloroplast genomes, and designed taxon-specific DNA mini-barcodes for the identification of processed medicines or canned food with degraded DNA. A 26-bp nucleotide signature was developed to find that Schisandrae Chinensis Fructus (Wuweizi in Chinese) was adulterated in CPMs (19). In this study, 12 batches of commercial CPMs from different manufacturers were investigated. The DNA mini-barcode of 159 bp, amplified with the specific primers 4F/4R for *Panax*, was utilized for the authenticity identification of the main herbal ingredient *P. ginseng* in Qipi pills, and the adulteration and substitution were successfully detected combining with double peak method. Finally, it was found that in three of the 12 batches of Qipi pills, *P. ginseng* was completely replaced or partially adulterated by *P. quinquefolius*. It can be predicted that in the future DNA mini-barcoding technology will play an indispensable role in the quality control of CPMs. And in the study, the mutation sites within the mini-barcode regions can also provide marker loci of KASP assay for the quality control of mass products.

### Development of KASP Technology Combined With DNA Mini-Barcode in the Large-Scale Market Supervision of CPMs

With the increasing recognition in disease treatment and prevention in public health, traditional Chinese medicine has gradually expanded into an international industry. In 2017, the global market economy of Chinese medicines reached 130 billion US dollars, which was expected to increase dramatically to 270 billion dollars by 2027; in 2019, the total trade volume of Chinese medicine products in China was 6.174 billion dollars, and the export volume was 4.019 billion dollars with an increasing trend (37). The products involved mainly include extracts, medicinal herbs, decoction pieces, CPMs and dietary supplementary products. In this huge market system of Chinese medicines, necessary quality control of large quantities of commodities under the market supervision is inevitable. In this context, a fast and accurate analysis method is urgently needed. KASP assay represents a new approach of genotyping, which can identify InDels and SNPs at specific genomic loci. It can be used for the simultaneous detection of a large number of samples with high throughput and low price, greatly improving the test efficiency. So far, KASP markers have been widely used in the fields of genotyping, molecular breeding and species identification (38). The core step of KASP technique is to select suitable gene loci as molecular markers for genotyping. In the present study, the two stable SNP sites found in the DNA mini-barcode region of *P. ginseng* and *P. quinquefolius* provided the marker loci of KASP analysis to investigate the authenticity and adulteration in CPMs. The fluorescence signals at mutation sites reflected the herbal composition in the 12 batches of CPM products. It is agreeable that the results of KASP detection were consistent with those obtained by Sanger sequencing, indicating the excellent accuracy and reliability of this method. Therefore, the KASP technology integrating with DNA mini-barcodes possesses great application potential in the large-scale market supervision of CPMs.

### More Attention Should Be Paid to the Pesticide Residues in CPMs

To cope with diseases and insect pests, various pesticides are often applied to medicinal plants to ensure their normal growth (39, 40). Yet the long-term and frequent supplement has led to the accumulation of pesticides in herbs, especially for the perennials. Even some banned pesticides are still used illegally, which may pose a great threat to human health (41). The amount of paclobutrazol in Ophiopogonis Radix was found to exceed the recommended maximum residue limits (42). Chlorpyrifos was frequently detected in Citri Reticulatae Pericarpium, Crataegi Fructus, and Cuscutae Semen (43). The unannounced inspections of Health Canada on medical cannabis products found that 26 of the 144 samples contained unauthorized pest control products (44, 45). The current focus is mainly on the pesticide residues in medicinal herbs and decoction pieces, while the possible adverse consequences caused by pesticides in CPMs are ignored. The Chinese pharmacopeia 2020 has also set the standards of pesticides in decoction pieces, but does not specify that in CPMs. GC-MS/MS is an effective method for the quantitation of trace chemical components (46). It has been widely used in the determination of pesticide residues at low concentrations owing to its high sensitivity (47–49). In this study, four kinds of pesticides were quantified by GC-MS/MS to evaluate the safety of CPMs. The results showed that at least one pesticide was detected in each of the 12 batches of CPM products. The HCH, which in China had been banned from its production and usage, was found in four of the samples. Moreover, the content of PCNB in the CPMs QPW6, QPW8, and QPW10 obviously exceeded the maximum limit of that in decoction pieces set by Chinese Pharmacopeia 2020, which may cause negative impact on their clinical safety. Therefore, our findings remind us of the necessity to establish a pesticide residues monitoring standard for CPMs.

### Significance of Multi-Method Combination in Quality Evaluation of CPMs

CPM is an important part of the medical and health system, which plays a key role in the prevention and treatment of diseases and health care. However, in contrast to single-component chemical drugs, the factors affecting the quality and efficacy of CPMs are more complex. On one hand, the pharmaceutical effect of CPMs is primarily affected by the content of active ingredients, the authenticity of raw materials, dosage forms, etc. On the other hand, the toxic and harmful substances in herbs, such as pesticides and heavy metals, are brought into the preparations, which may adversely affect the clinical safety of CPMs. During the National Drug Sampling Inspection of China in 2020, a total of 5,842 batches of CPMs were investigated; the main problems found in CPMs included raw material adulteration, mycotoxin residues, and excessive pesticides and heavy metals (50). In this context, single method such as microscopic observation or chemical assay, is unable to carry out a comprehensive evaluation of proprietary Chinese medicines. Contrarily, the combination of multiple methods shows greater advantages and practical value. In the present study, KASP technology and GC-MS/MS were combined to investigate the herbal composition and pesticide residues of 12 commercially available CPMs, and to comprehensively assess their safety and effectiveness. The forbidden pesticide and adulteration of raw materials were found to coexist in two batches, which undoubtedly led to a decrease in both medical efficacy and clinical safety. Hence, this study further demonstrates that it is essential to establish a comprehensive evaluation of CPMs by multi-index detection with the combination of multiple methods.

## Conclusion

In this study, KASP technology cooperating with DNA mini-barcoding and GC-MS/MS were utilized to investigate the herbal components and pesticide residues of 12 batches of commercially available CPMs. Adulteration and substitution of raw materials were identified in CPMs. PCNB was frequently detected and the banned pesticide HCH was also found. Two batches of the products were found to contain both adulteration and prohibited pesticide. Our results suggest that KASP technology combined with DNA mini-barcoding possesses great potential in the quality assessment of CPMs during the large-scale market supervision. This study provides a baseline reference for the comprehensive evaluation of proprietary Chinese medicine by multi-method combination, and shows a deeper insight into the pesticide residues in CPMs.

## Data Availability Statement

The original contributions presented in the study are included in the article/Supplementary Material, further inquiries can be directed to the corresponding author.

## Author Contributions

JH: conceptualization. GW and XB: experiment and data analysis. GW, XB, and YR: resources. GW: writing—original draft. XC, JH, and XP: writing-review & editing. All authors contributed to the article and approved the submitted version.

## Funding

This work was supported by the National Key Research and Development Program of China (2019YFC1604701).

## Conflict of Interest

The authors declare that the research was conducted in the absence of any commercial or financial relationships that could be construed as a potential conflict of interest.

## Publisher's Note

All claims expressed in this article are solely those of the authors and do not necessarily represent those of their affiliated organizations, or those of the publisher, the editors and the reviewers. Any product that may be evaluated in this article, or claim that may be made by its manufacturer, is not guaranteed or endorsed by the publisher.
