# Supplementary material for: Detection of Adulteration and Pesticide Residues in Chinese Patent Medicine Qipi Pill Using KASP Technology and GC-MS/MS
Source: Front Nutr. 2022 Mar 10;9:837268. doi: 10.3389/fnut.2022.837268 (PMC8965643; doi:10.3389/fnut.2022.837268)
Supplement: Supplementary file 1 [file Image_1.pdf]

Supplementary material:

Figure S1. GC-MS/MS chromatogram of 12 batches of Qipi Pill for pesticide residue detection.

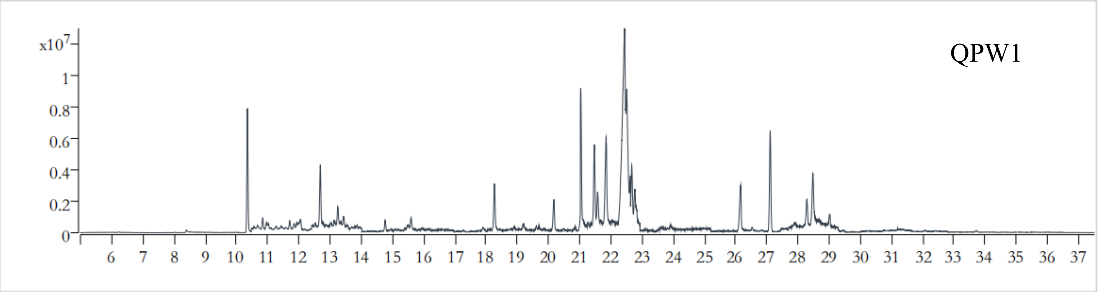

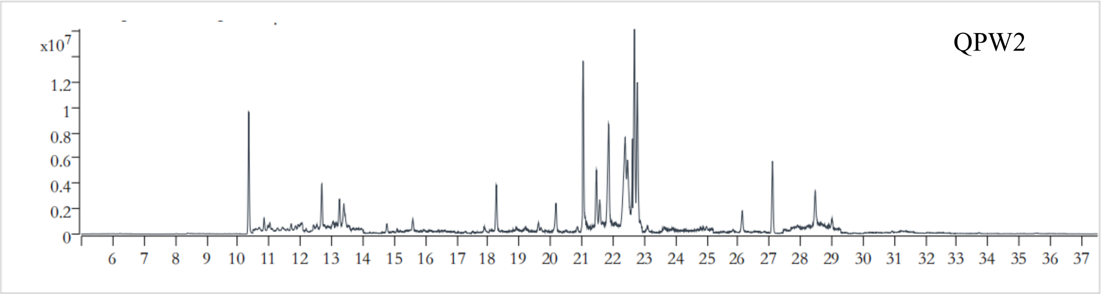

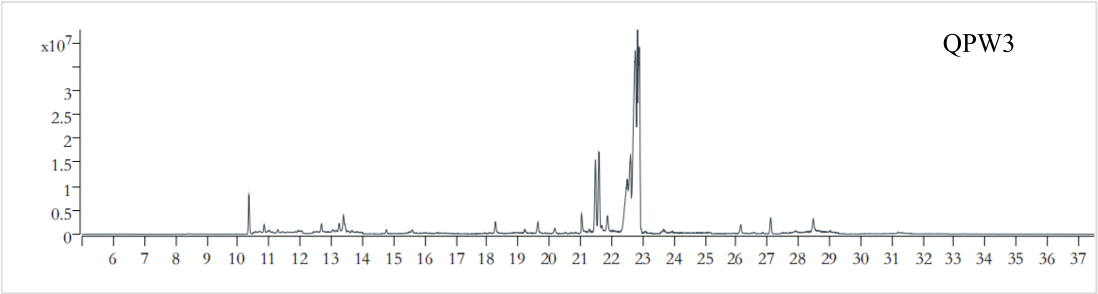

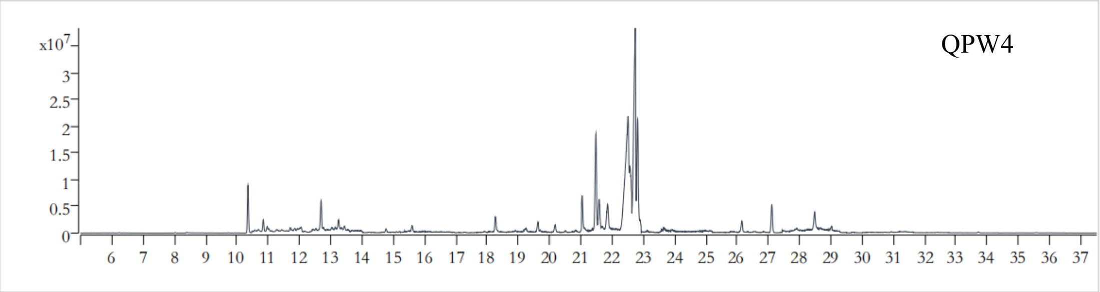
QPW4
x10^7
0 0.5 1 1.5 2 2.5 3
6 7 8 9 10 11 12 13 14 15 16 17 18 19 20 21 22 23 24 25 26 27 28 29 30 31 32 33 34 35 36 37

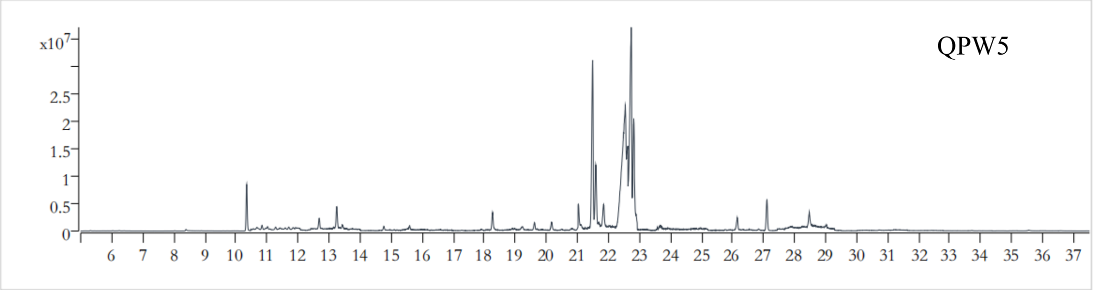
QPW5
x10^7
0 0.5 1 1.5 2 2.5
6 7 8 9 10 11 12 13 14 15 16 17 18 19 20 21 22 23 24 25 26 27 28 29 30 31 32 33 34 35 36 37

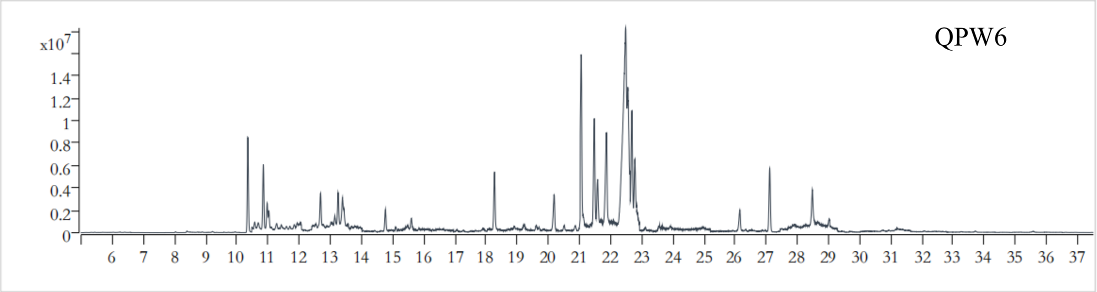
QPW6
x10^7
0 0.2 0.4 0.6 0.8 1 1.2 1.4
6 7 8 9 10 11 12 13 14 15 16 17 18 19 20 21 22 23 24 25 26 27 28 29 30 31 32 33 34 35 36 37

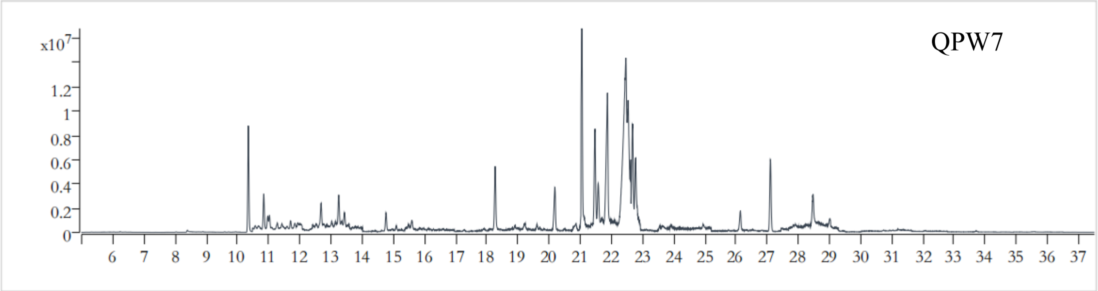
QPW7
x10^7
0 0.2 0.4 0.6 0.8 1 1.2
6 7 8 9 10 11 12 13 14 15 16 17 18 19 20 21 22 23 24 25 26 27 28 29 30 31 32 33 34 35 36 37

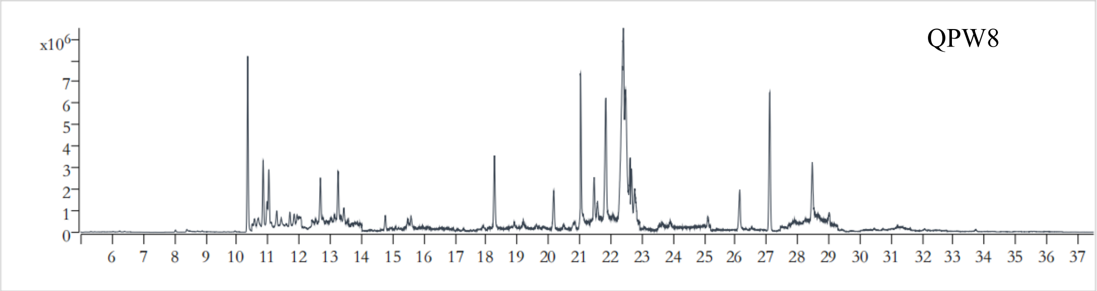
QPW8
x10^6

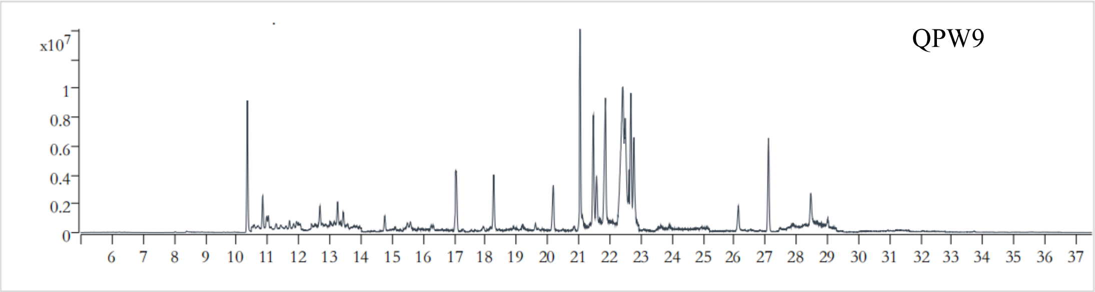
QPW9
x10^7

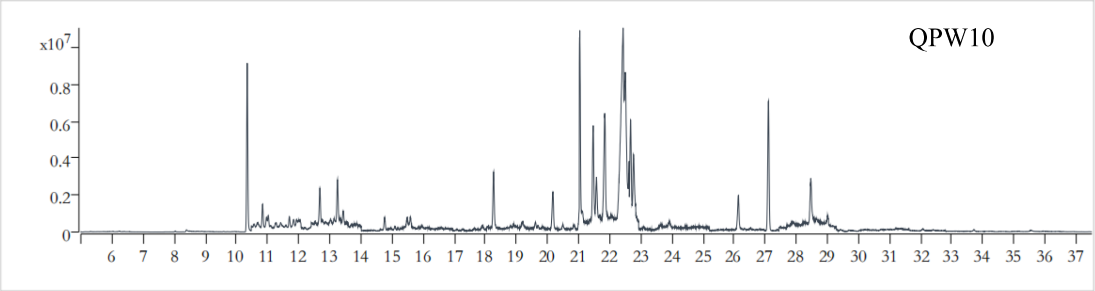
QPW10
x10^7

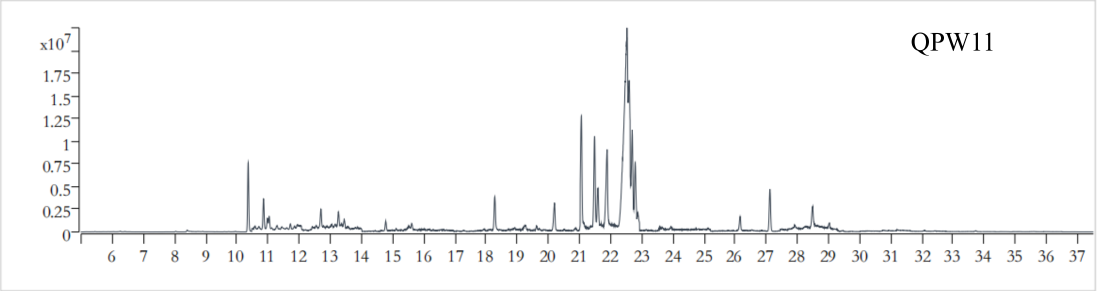
QPW11
x10^7

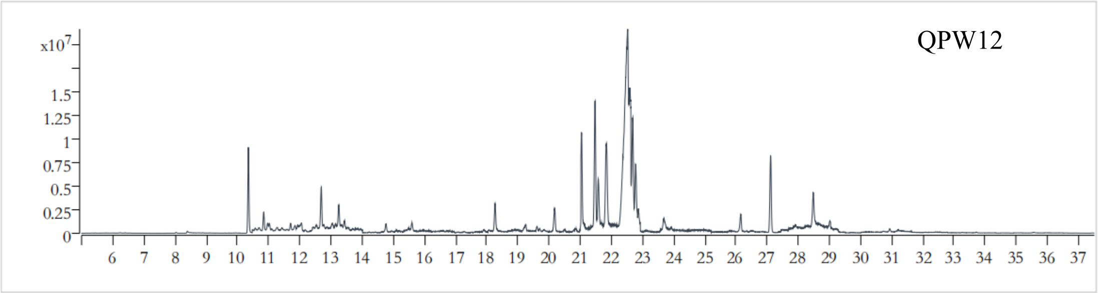

Figure S1. GC-MS/MS chromatogram of 12 batches of Qipi Pill for pesticide residue detection.
